# Giant 20 × 35 cm brachial artery pseudoaneurysm after fistulogram treated with surgical resection of pseudoaneurysm and patch angioplasty of brachial artery

**DOI:** 10.1093/jscr/rjae213

**Published:** 2024-04-02

**Authors:** Naveed A Rahman, Alice Wang, Deena B Chihade, Anthony Feghali

**Affiliations:** Department of Surgery, SUNY Upstate Medical University, 750 East Adams Street, Syracuse, NY 13210, United States; Department of Surgery, SUNY Upstate Medical University, 750 East Adams Street, Syracuse, NY 13210, United States; Department of Surgery, SUNY Upstate Medical University, 750 East Adams Street, Syracuse, NY 13210, United States; Department of Surgery, SUNY Upstate Medical University, 750 East Adams Street, Syracuse, NY 13210, United States

**Keywords:** brachial, pseudoaneurysm, giant, fistulogram, dialysis

## Abstract

Brachial artery pseudoaneurysms are a rare entity, which can occur secondary to infectious, traumatic, or iatrogenic causes. We present a 78-year-old female with end-stage renal disease on hemodialysis via a right brachio-basilic arteriovenous fistula. She had previously undergone numerous fistulograms and endovascular interventions for right upper extremity swelling due to prolonged bleeding following dialysis. After a recent fistulogram she developed recurrent arm swelling. Duplex showed a large hematoma without any evidence of vascular flow. However, intraoperatively, she was noted to have a giant 20 × 35 cm pseudoaneurysm of the brachial artery. Therapeutic options include endovascular stenting, embolization, thrombin injection, ultrasound-guided compression, and surgery. We elected to perform resection of the large pseudoaneurysm and arteriovenous fistula ligation due to the large size. Given her end-stage renal disease status and lacking quality autogenous vein, we were able to perform a patch angioplasty repair of her brachial artery without requiring a bypass.

## Introduction

A pseudoaneurysm (PSA), or false aneurysm, is a pulsatile hematoma that is formed secondary to hemorrhage from an artery contained by the soft tissues and fascia [1]. PSAs can grow or even rupture due to the pressure of pulsatile blood flow exceeding the compliance of the surrounding tissue. They are most commonly discovered within the lower extremity vasculature [2]. Brachial artery PSAs are rare entities which can occur secondary to traumatic causes or recurrent arterial puncture [1]. Risk factors include large sheath size, faulty puncture technique, hypertension, heavily calcified arteries, and hemodialysis [3]. Consequences associated with PSAs are rupture, infection, compartment syndrome, and local skin ischemia [1]. We report a case of brachial artery PSA after numerous access attempts for hemodialysis and fistuolograms. The patient passed away from unrelated causes in 2021 and her daughter provided publication consent.

## Case report

**Figure 1:**
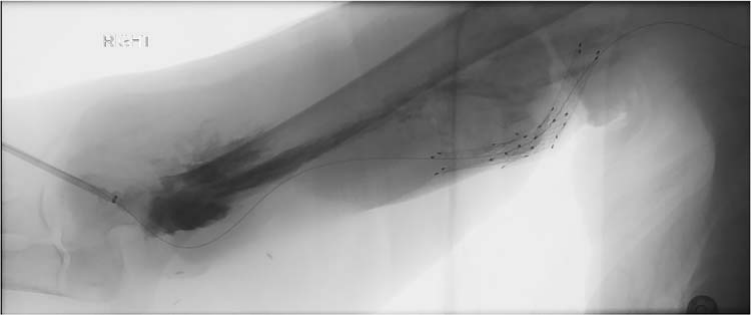
Fistulogram demonstrating contrast extravasation around the sheath access site.

A 78-year-old female with history of coronary artery disease, hypertension, hyperlipidemia, and end-stage renal disease on hemodialysis through a right brachio-basilic arteriovenous fistula presented to the hospital with prolonged bleeding after dialysis. She was not taking any antiplatelet or anticoagulation medication. She had previously undergone stenting of the proximal basilic vein due to venous stenosis. Right upper extremity fistulogram identified recurrent basilic vein stenosis within the previous stents. Balloon venoplasty was performed via 6 Fr sheath. Repeat fistulogram demonstrated persistent stenosis of the proximal basilic vein and so the sheath was upsized for Viabahn stent placement. During the sheath exchange, there was some contrast extravasation around the sheath access site (Fig. 1), which resolved with a longer 7 Fr sheath. Completion fistulogram did not show residual stenosis and the patient was discharged the following day. The patient continued to use her fistula for hemodialysis during the next 2 months.

**Figure 2:**
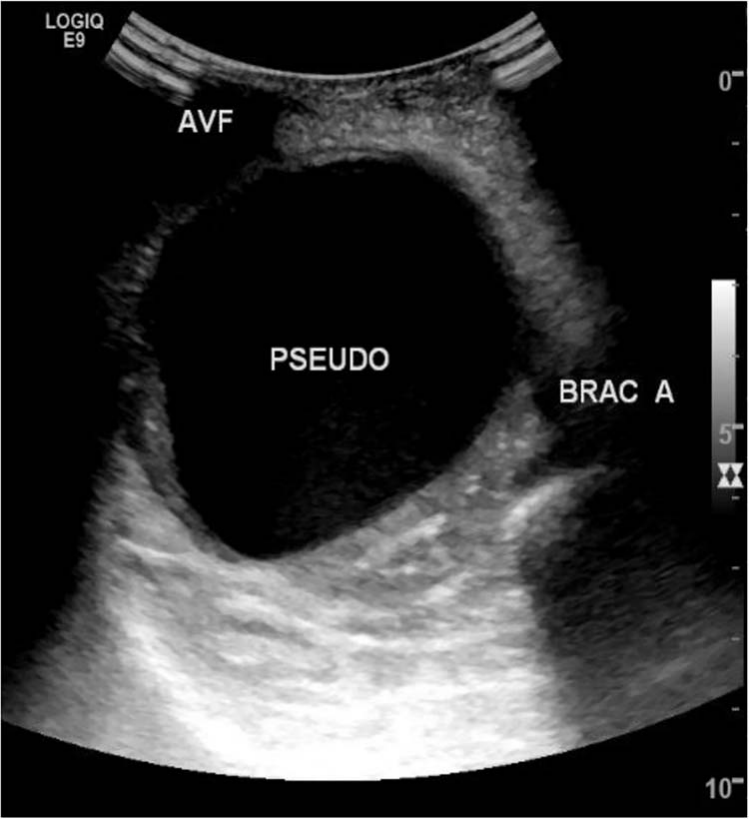
Duplex ultrasound of brachial artery pseudoaneurysm.

**Figure 3:**
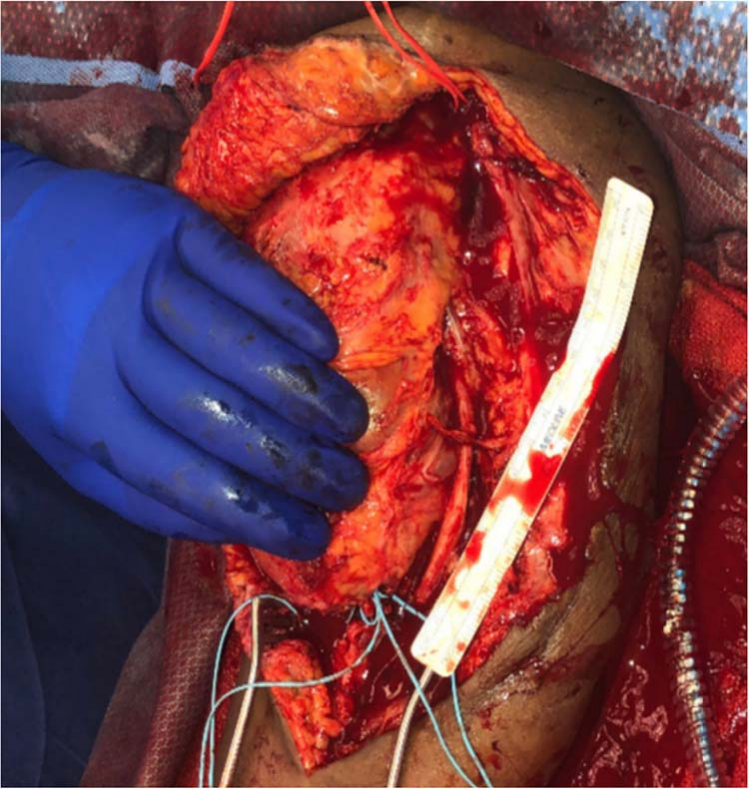
20 × 35 cm brachial artery pseudoaneurysm.

**Figure 4:**
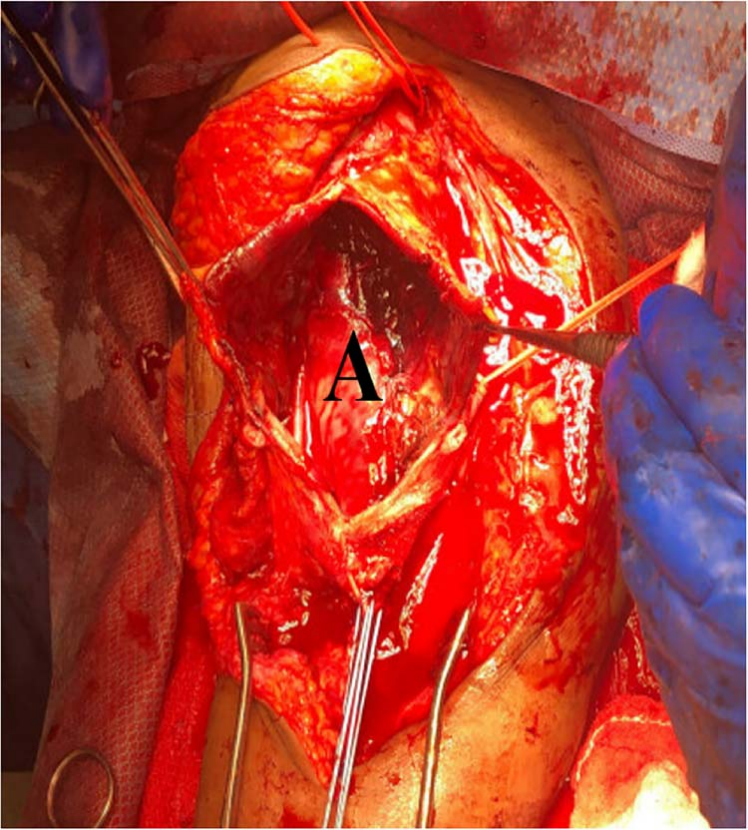
Decompression of giant brachial artery pseudoaneurysm.

**Figure 5:**
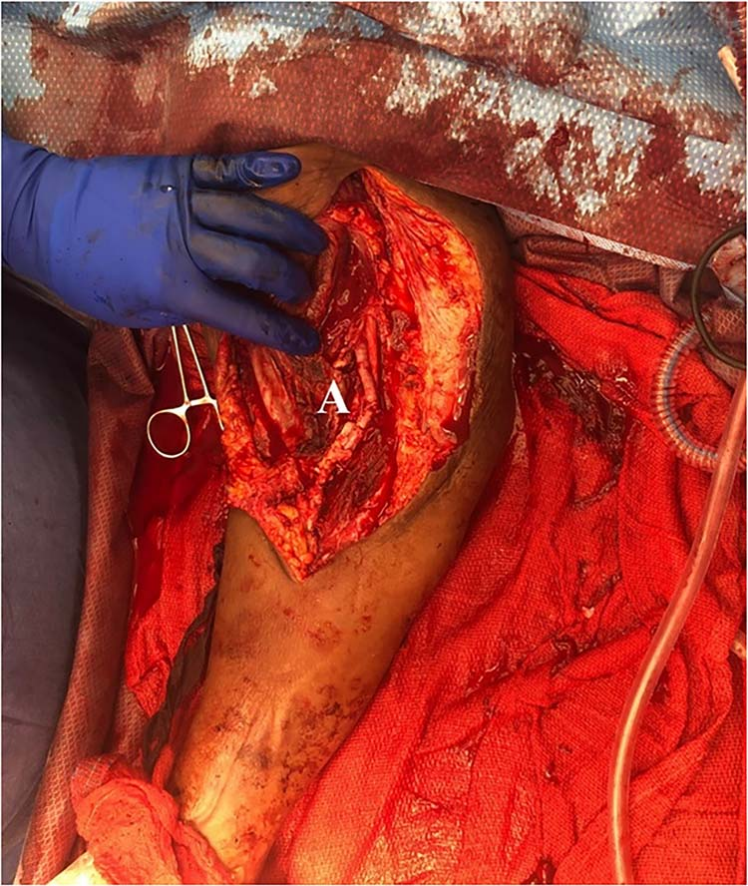
Completed patch angioplasty of brachial artery (A).

She then re-presented 2 months later with gradually increasing right upper extremity edema with a palpable thrill in the arteriovenous fistula. Duplex identified a large hematoma with patency of the fistula (Fig. 2). A tunneled dialysis catheter was placed to allow the swelling and hematoma to resolve. After medical optimization, the patient consented for operative intervention with the intention to evacuate the hematoma. There was noted to instead be a 20 × 35 cm PSA of the brachial artery (Fig. 3). The median nerve was identified and preserved. After proximal and distal control, the patient was heparinized, and the brachial artery PSA was decompressed with evacuation of large mural thrombi (Fig. 4). This artery segment was resected, and patch angioplasty was performed (Fig. 5). The fistula was ligated, and the distal remnant was preserved. Radial and ulnar pulses were palpable. The patient recovered well postoperatively.

## Discussion

Arteriovenous access hematomas are not uncommon but the presentation of a large brachial artery pseudoaneurysm is very rare. Pseudoaneurysms can be congenital, associated with connective tissue disorders, or due to trauma or iatrogenic causes [2]. Brachial PSAs are rare and occur in <0.04% of cases, but incidence is rising with increased brachial endovascular access [4]. They are estimated to be formed by iatrogenic causes occurring in 0.3% to 0.7% of patients [5, 6]. In our case, the PSA may have been formed during an inadvertent arterial puncture site from dialysis or during previous fistulograms. The PSA was initially assumed to be a hematoma as prior duplex did not show flow into the cavity.

Therapeutic options for PSAs are dependent on criteria such as size, location, accessibility, presence of a neck, and pathogenesis. There are several treatment modalities including endovascular stenting, embolization, thrombin injection, ultrasound-guided compression (USGC), and surgery. Endovascular stent-graft implants have success rates of 88.3% in post-catheterization arterial femoral PSAs and 92% in hepatic artery PSA exclusion but are expensive and carry the possibility of device failure [7, 8]. A study by Yetkin *et al*. [9] recommends using endovascular polytetrafluoroethylene-covered stent grafts because of success with treating various peripheral PSAs. Embolization is indicated for PSAs arising from small brachial artery branches. However, if there is collateral circulation that supplies blood to the distal tissues, brachial artery embolization may be performed [10].

Percutaneous thrombin injection has favorable outcomes and low recurrence rate but carries the risk of distal embolization, necrosis, abscess formation, and PSA rupture [2]. Small PSAs can be treated noninvasively with procedures such as USGC, which involves applying pressure with an ultrasound transducer over the neck of the PSA until blood flow is stopped. The brachial artery is difficult to compress due to its mobility and the shape of the humerus and this procedure can be quite painful [4].

Surgical measures in treating PSAs include resection, ligation, re-anastomosis, or vein graft interposition. Open surgery is indicated with rapidly expanding aneurysms, larger PSA size, rupture, distal ischemia, or neuropathy of the median nerve from local pressure [11]. Smaller arterial defects can be repaired with sutures or patch angioplasty [12]. Axillo-radial or axillo-ulnar bypass grafting with reversed great saphenous vein can be considered [9]. Saphenous vein interposition graft is preferred when the PSA is located at or proximal to the brachial bifurcation in order to maintain continuity and extremity viability [9]. PSA sac excision may also be performed with vascular continuity maintained with an end-to-end anastomosis between the brachial artery and interposition vein bypass graft [13].

We elected to perform pseudoaneurysmal resection and arteriovenous fistula ligation due to the large pseudoaneurysmal size. Often, patients with end-stage renal disease also lack quality autogenous vein for interposition grafting. Primary repair with bovine patch can be performed immediately without additional vein harvest time and is a viable option to avoid interposition grafting.

To prevent the formation of iatrogenic PSAs in the future, dialysis centers should be advised on the needle gauge used, access location, and appropriate pressure control after each session. The most common cause of brachial artery pseudoaneurysm of arteriovenous fistulas is repeated cannulation during dialysis sessions, occurring in 1 in 13 000 hemodialysis sessions [14].

However, this is the first case report describing a truly giant, > 20 × 35 cm, brachial artery pseudoaneurysm from a dialysis patient. Duplex can help evaluate dialysis issues, but as demonstrated by this case, may not accurately identify all access complications or hematomas.

## Notes

Paper presented at: Eastern Vascular Society 36th Annual Meeting, Philadelphia, PA, Sept 29-Oct 1, 2022
